# Aerobic exercise does not affect serum neurofilament light in patients with mild Alzheimer’s disease

**DOI:** 10.3389/fnins.2023.1108191

**Published:** 2023-01-24

**Authors:** Kristian Steen Frederiksen, Camilla Steen Jensen, Peter Høgh, Robert Gergelyffy, Gunhild Waldemar, Birgitte Bo Andersen, Hanne Gottrup, Karsten Vestergaard, Lene Wermuth, Helle Bach Søndergaard, Finn Sellebjerg, Steen Gregers Hasselbalch, Anja Hviid Simonsen

**Affiliations:** ^1^Department of Neurology, Danish Dementia Research Center, Copenhagen University Hospital–Rigshospitalet, Copenhagen, Denmark; ^2^Department of Neurology, Regional Dementia Research Centre, Zealand University Hospital, University of Copenhagen, Copenhagen, Denmark; ^3^Department of Clinical Medicine, Regional Dementia Research Centre, Zealand University Hospital, University of Copenhagen, Copenhagen, Denmark; ^4^Department of Geriatrics, Slagelse Hospital, Slagelse, Denmark; ^5^Department of Clinical Medicine, Faculty of Health and Medical Sciences, University of Copenhagen, Copenhagen, Denmark; ^6^Dementia Clinic, Aarhus University Hospital, Aarhus, Denmark; ^7^Dementia Clinic, Aalborg University Hospital, Aalborg, Denmark; ^8^Department of Neurology, Dementia Clinic, Slagelse Hospital, Slagelse, Denmark; ^9^Department of Regional Health Research, University of Southern Denmark, Odense, Denmark; ^10^Danish Multiple Sclerosis Center, Copenhagen University Hospital–Rigshospitalet, Glostrup, Denmark

**Keywords:** exercise, Alzheimer’s disease, dementia, intervention, neurofilament light, neurodegeneration

## Abstract

**Introduction:**

Aerobic exercise has been shown to modify Alzheimer pathology in animal models, and in patients with multiple sclerosis to reduce neurofilament light (NfL), a biomarker of neurodegeneration.

**Objective:**

To investigate whether a 16-week aerobic exercise program was able to reduce serum NfL in patients with mild Alzheimer’s disease (AD).

**Methods:**

This is a secondary analysis of data from the multi-center Preserving Cognition, Quality of Life, Physical Health, and Functional Ability in Alzheimer’s disease: The Effect of Physical Exercise (ADEX) study. Participants were randomized to 16 weeks of moderate intensity aerobic exercise or usual care. Clinical assessment and measurement of serum NfL was done at baseline and after the intervention.

**Results:**

A total of 136 participants were included in the analysis. Groups were comparable at baseline except for *APOE*ε*4* carriership which was higher in the usual care group (75.3 versus 60.2%; *p* = 0.04). There was no effect of the intervention on serum NfL [intervention: baseline NfL (pg/mL) 25.76, change from baseline 0.87; usual care: baseline 27.09, change from baseline −1.16, *p* = 0.09].

**Conclusion:**

The findings do not support an effect of the exercise intervention on a single measure of neurodegeneration in AD. Further studies are needed using other types and durations of exercise and other measures of neurodegeneration.

**Clinical trial registration:**

clinicaltrials.gov, identifier NCT01681602.

## Introduction

Physical activity is a prerequisite for the maintenance of health and wellbeing in humans (Penedo and Dahn, 2005). Moreover, physical activity is a protective factor in terms of preventing cognitive decline including dementia (Livingston et al., 2017) as indicated by findings from a number of epidemiological studies (Wang et al., 2022). Alzheimer’s disease (AD) is a common cause of cognitive decline and is neuropathologically characterized by accumulation of beta-amyloid and tau upstream from neurodegenerative changes in the pathophysiological cascade (Selkoe and Hardy, 2016). Such changes may be measured by Magnetic Resonance Imaging (MRI) or fluid biomarkers including Neurofilament Light (NfL). NfL is released into the Cerebrospinal Fluid (CSF) from damaged neurons and may be detected in serum (Molinuevo et al., 2018). NfL is elevated in a number of conditions such as AD and ischemic stroke. In AD, NfL increases as the disease progresses (Palmqvist et al., 2019). At present, NfL measured in CSF has been used as a secondary outcome measure in AD trial of potential new drugs (Li et al., 2022), whereas measurements in serum is less well established. Physical exercise may also be able to ameliorate symptoms of AD such as cognitive symptoms and behavioral changes (Hoffmann et al., 2013; Groot et al., 2016). In the ADEX study, we found that 16 weeks of exercise improved processing speed and measures of physical function and fitness (Hoffmann et al., 2013) and that this effect may be larger with the Apolipoprotein E (*APOE)* ε4 genotype. Further, exercise did not affect beta-amyloid accumulation either on Positron Emission Tomography (Frederiksen et al., 2019b)or in CSF (along with measures of total tau and phosphorylated tau) (Jensen et al., 2016). In transgenic AD mouse models, exercise has been reported to attenuate hallmarks of AD pathology such as beta-amyloid (Vasconcelos-Filho et al., 2021) and tau pathology (Belarbi et al., 2011). This demonstrates that exercise might engage targets relevant for a disease-modifying effect in AD. However, studies so far in humans have not been able to substantiate a comparable effect. For example, despite early and promising findings on the effects of exercise on hippocampal volume (Erickson et al., 2011) and cortical thickness in healthy older adults (Colcombe et al., 2006), subsequent studies with varying durations of interventions from 3 to 24 months have been disappointing (Firth et al., 2018; Wilckens et al., 2021), including in AD patients (16 week intervention) (Frederiksen et al., 2017, 2018). Data from human studies are more sparse regarding effects on tau and beta-amyloid, but so far evidence to suggest that results from animal studies extends to humans, is missing (Frederiksen et al., 2017, 2019a,b). Other factors might explain the lack of an association. For example, many studies have used brain MRI scans to measure effect on neurodegeneration (Frederiksen et al., 2017, 2018; Broadhouse et al., 2020), which may not be sensitive enough to pick up small effects, at least in terms of structural changes such as changes in cortical thickness and hippocampal volume. Although this remains speculative, findings in multiple sclerosis demonstrate that exercise interventions of a short duration clearly attenuate measures related to neurodegeneration in a similar fashion as disease-modifying therapy. Specifically, in two studies in multiple sclerosis exercise interventions were able to modify serum concentrations of neurofilament light chain (NfL) (Ercan et al., 2021; Joisten et al., 2021). Studies in AD on NfL (in serum or CSF) in blood and exercise are sparse. A single study reported changes over a 6-month period in plasma NfL in a small sample of AD patients. Patients were either randomized to cycling or stretching. No significant change over time was reported and between-group differences were not reported (Raket et al., 2020).

The effects of exercise may be dependent on *APOE* ε4 genotype, a risk allele for AD. In a study, we found that AD patients who were carriers of the *APOE*-ε4 allele benefitted more from a 16-week exercise intervention in regards to cognitive, neuropsychiatric, and physical performance (Jensen et al., 2019b). Epidemiological studies have also found evidence of a differential effect of physical activity levels depending on *APOE*-ε4 status with regards to cognitive decline and hippocampal volume (Smith et al., 2014).

As the measurement of NfL in serum could be a particularly sensitive biomarker of neurodegeneration, the aim of the present study was to assess the effects of a physical exercise intervention on serum NfL in patients with AD. In addition, we wished to explore the influence of *APOE* status on the effects of the intervention on NfL.

## Materials and methods

### Study design and population

**FIGURE 1:**
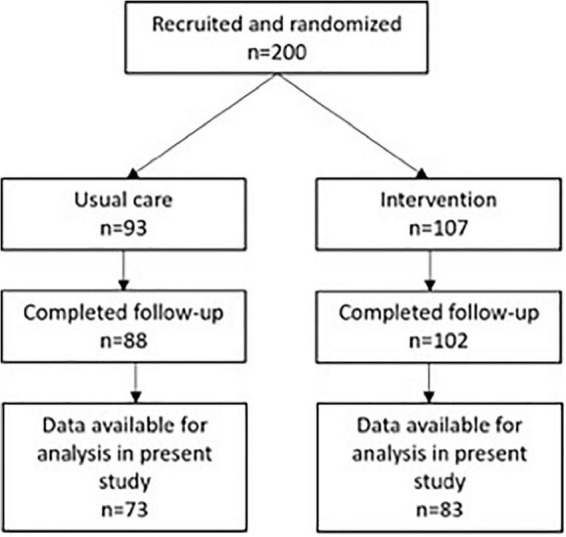
Flow-chart for participants. Data available for analysis in present study refers to availability of serum neurofilament light at both baseline and follow-up.

The present study is a secondary analysis of data from the Preserving Cognition, Quality of Life, Physical Health, and Functional Ability in Alzheimer’s Disease: The Effect of Physical Exercise (ADEX) study, a multi-center, single-blinded randomized controlled trial (RCT) of physical exercise in patients with mild AD. Details of the rationale and study design can be found elsewhere (Hoffmann et al., 2013, 2016), as well as the main findings (Hoffmann et al., 2013, 2016). The intervention comprised 16 weeks of group-based, supervised moderate- to high-intensity aerobic exercise three times weekly for 1 h. An initial 4-week ramp-up period which familiarized participants with the exercise equipment and focused on strength exercises was conducted to reduce the risk of injuries (Hoffmann et al., 2013, 2016). Two hundred participants from eight centers were recruited and randomized in the study. Clinical assessment of cognitive function, activities of daily living, physical function, aerobic fitness, neuropsychiatric symptoms at baseline and at follow-up immediately following the end of the 16-week intervention was carried out. All assessments were performed by assessors blinded to group allocation. Inclusion criteria for the study were the following: (1) AD according to National Institute of Neurological and Communicative Diseases and Stroke/Alzheimer’s Disease and Related Disorders Association (NINCDS-ADRDA) Alzheimer’s Criteria and DIagnostic and Statistical Manual (DSM)-IV codes; (2) between 50 and 90 years of age; (3) a Mini–Mental State Examination score of more than 19; (4) at least 3 months of stable doses if receiving anti- dementia medication or mood-stabilizing medication; and (5) informed consent. Exclusion criteria included the following: (1) severe psychiatric illness; (2) alcohol or drug abuse within the last 2 years; (3) participation in aerobic exercise (moderate to high intensity) more than twice weekly on a regular basis; and (4) any medical condition precluding participation in the exercise program (e.g., severe neurological or medical illness and presence of several cardiovascular risk factors). Out of the 200 participants in the study, 156 had data available for the present study and were included in the analysis (Figure 1). The ADEX trial was approved by the Committees of Biomedical Research Ethics for the Capital Region (protocol no.: H-3-2011-128) and by the Danish Data Protection Agency (file no.: 30-0718) and carried out in accord with the Helsinki Declaration of 1975. The trial was registered at clinicaltrials.gov (identifier: NCT01681602) on September 10, 2012.

### Measurement of NfL

Neurofilament light concentrations in serum were measured using the SIMOA^®^ NF-light™ Advantage Kit (Reference number 103186) (Quanterix, Lexington, MA, United States) and the manufacturer’s instructions. Measurements were performed on a Quanterix SR-X Biomarker Detection System.

### APOE genotype

DNA was isolated with Promega Maxwell DNA purification kits (Promega, WI, USA), according to the manufacturer’s protocol from 250 μL of buffy coat from 6 mL Ethylenediaminetetraacetic acid (EDTA) vials. *APOE* genotyping for the ε2, ε3, and ε4 alleles was performed with a TaqMan qPCR assay as described by Koch et al. (2002).

### Clinical scales and measures

Cognitive assessment included the MMSE for global cognitive impairment (Folstein and Folstein, 1975), the Symbol Digit Modalities Test (SDMT) after 120 s for mental speed and attention (Smith, 1973). Assessment of Activities of Daily Living (ADL) functioning was assessed using the AD Cooperative Study-ADL scale (Galasko et al., 1997), and is reported for baseline. Behavioral and psychological symptoms of dementia were assessed at baseline using the Neuropsychiatric Inventory–Questionnaire (NPI-Q) (Kaufer et al., 2000).

### Assessment of physical fitness

Test of Physical Performance The 6-min Astrand Cycle Ergometer test (Monark Ergomedic 839E; Monark Exercise AB, Sweden) was used to estimate the maximal oxygen uptake based on workload and average heart rate (HR) during the last minute of the 6-min cycle test, corrected for age and body weight. Maximal oxygen uptake (estimated VO2 max) was used as a measure of aerobic, physical fitness (Cink and Thomas, 1981). Timed Up and Go test (TUG) was used to assess basic mobility by measuring the time it takes for a person as quickly and safely to stand up from a chair, walk 3 m, cross a line, turn around, and walk back to the chair and sit down (Podsiadlo and Richardson, 1991). The 30-s chair stand test (STS) assesses strength and endurance in the lower extremity by measuring the number of stands completed in 30 s with hands crossed against the chest (Eggermont et al., 2010). The 400-m walk test (fast gait speed) was used to assess walking endurance. The walking time(s) was measured on a 20-m course marked with two colored cones (Guralnik et al., 1994). The 10-m walk test (usual gait speed) assesses usual gait speed (m/s) on a 10-m long course (Maquet et al., 2010).

### Statistical analysis

Baseline characteristics were compared between the two groups using Student’s un-pairs samples *t*-test for continuous variables and the chi-squared test for categorical variables. Regarding the analysis of the effects of the intervention, we first tested whether the previously reported effect of the exercise on VO2max was also present in this subgroup as an effect on NfL could possibly be mediated through an effect on aerobic capacity. This was done using Student’s un-paired samples *t*-test for change in VO2max from baseline to follow-up and was also done for all other measures included in correlation analyses. The effect of aerobic exercise on serum NfL was assessed by ANalysis of COVAriance (ANCOVA) with change in serum NfL from baseline to follow-up as dependent variables and group allocation, baseline MMSE, APOE genotype and sex, and APOE genotype x group allocation as covariates. Subgroup analyses for the analysis of the main effects of the intervention of NfL concentration was carried out in those participants who exercised with a mean intensity of >70% of maximal HR and attended >80% of the offered sessions (high exercisers) or below (low exercisers). As a sensitivity analysis and to further explore the effects of *APOE* ε4 status, *post hoc* Spearman’s correlations were calculated across numbers of alleles and change from baseline to follow-up in NfL concentrations.

To test a possible association between improvements in physical fitness and serum NfL, we further examined whether there was a correlation between change in estimated VO2max and change in serum NfL in the intervention group using Spearman’s rank correlation. Similarly, the relationship between change in the TUG, STS, 10-m, and 400-m walk tests and change in serum NfL were assessed. As the intervention has been shown to improve performance on the SDMT, we also assessed whether change in SDMT correlated with change in NfL. Statistical significance was set at P, 0.05 (two-tailed). Statistical analysis was carried out using Intercooled Stata 9.2 for Macintosh (Stata Corporation, USA).

## Results

**FIGURE 2:**
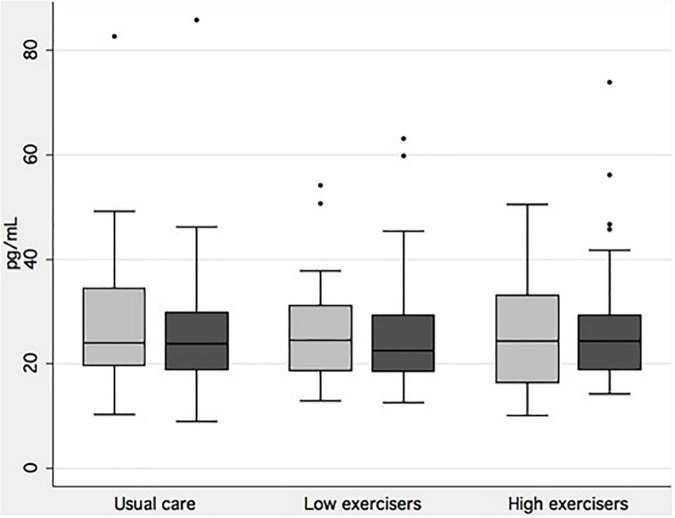
Serum neurofilament light by exercise intensity. Boxplot of baseline (light gray) and follow-up measurements of neurofilament light chain for each group of exercise intensity achieved in the intervention group, and for the usual care group. There were no significant differences in changes from baseline to follow-up between groups.

**FIGURE 3:**
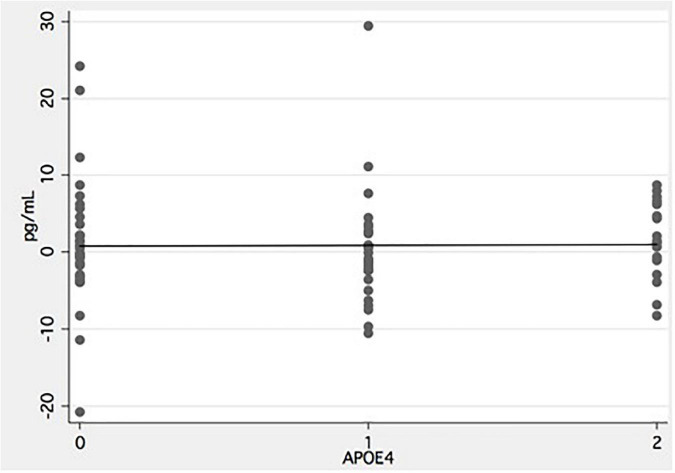
Effect of number of *APOE-*ε*4* alleles on neurofilament light. *X*-axis indicate number of APOE-ε4 alleles and *y*-axis change in serum concentration from baseline to follow-up in the intervention group. Negative values indicate a drop in serum NfL from baseline to follow-up.

Baseline demographics and clinical characteristics are presented in Table 1. There was a significant difference in the prevalence of the *APOE* ε4 allele with the allele being more frequent in the usual care group (75.3 versus 60.2%). A total of 20.7% of those in the intervention group and 23.9% of the usual care group had two *APOE* ε4 (not significant). There were no other significant differences in baseline characteristics between the two groups. There were no significant differences between the included study population and the study population comprised of dropouts and missing serum NfL values in any of the baseline variables (data not reported). There was no significant difference in change from baseline in VO2max between the two groups [intervention: 3.5 (SE 0.8); usual care: 2.2 (SE 0.6); *p* = 0.13] (Table 2). There was no effect of the intervention on serum NfL levels (Table 2) between groups [*F*(1,153) = 2.52, *p* = 0.11] (Table 2) or an interaction between APOE genotype or group allocation [*F*(1,153) = 1.01, *p* = 0.36]. Similarly, there was no difference in the effect of the intervention between the usual care group, low exercisers or high exercisers [usual care versus low exercisers: *F*(1,100) = 1.03, *p* = 0.31; usual care versus high exercisers *F*(1,119) = 2.80, *p* = 0.10] (see Figure 2). Correlation analyses of number of *APOE*- ε4 and change in NfL was not significant (see Figure 3). Lastly, there were no significant findings regarding correlations between change in NfL and change in physical fitness measures and SDMT (Table 3).

**TABLE 1:** Baseline demographics and clinical measures.

	Usual care group (n = 73)	Intervention group (n = 83)	*P*-value
Age [years (SD)]	70.6 (7.2)	69.5 (7.8)	0.39
Female participants [n (%)]	27 (38)	38 (46)	0.27
Baseline MMSE mean, (SD)	24.2 (3.9)	23.9 (3.4)	0.57
Baseline ADCS-ADL mean, (SD)	63.1 (10.5)	65.3 (8.7)	0.16
Baseline NPI-Q mean, (SD)	10.1 (10.2)	9.9 (10.5)	0.91
Baseline V_*O*2_ max (ml/kg) mean, (SD)	26.3 (9.2)	25.4 (7.8)	0.54
Baseline neurofilament light chain (pg/mL) mean, (SD)	27.1 (11.7)	25.8 (9.7)	0.44
APOE-ε4^#^	75.3%	60.2%	**0.04**
SDMT mean, (SD)	28.7 (14.5)	26.7 (15.1)	0.14
TUG (seconds) mean, (SD)	6.3 (1.74)	6.7 (1.63)	0.08
10 m walking speed (meter/second) mean, (SD)	7.7 (1.78)	8.0 (1.76)	0.12
400 m (seconds) mean, (SD)	297.9 (84.8)	306.9 (86.4)	0.26
STS (seconds) mean, (SD)	14.7 (4.7)	14.3 (3.6)	0.72

Table shows baseline characteristics by group allocation. Significant *p*-values indicated by bold lettering. All values given as mean (± standard deviation).

^#^Percentage of participants with at least one APOE-ε4 allele.

ADCS-ADL, Alzheimer’s disease cooperative study–activities of daily living; APO, apolipoprotein; MMSE, mini mental state examination; NPI-Q, neuropsychiatric inventory–questionnaire; SD, standard deviation; SDMT, symbol digit modalities test; STS, sit-to-stand; TUG, timed-up-and-go.

**TABLE 2:** Effects of the intervention.

	Usual care (n = 73)	Intervention (n = 83)	*P*-value
	Change from baseline	Change from baseline	
Neurofilament light chain (pg/mL), (SE)	−1.24 (0.86)	0.74 (0.69)	0.11
SDMT mean, (SE)	−1.8 (0.89)	−2.0 (0.82)	0.55
VO2 max (mL/kg) mean, (SE)	2.2 (SE 0.6)	3.5 (0.8)	0.13
TUG (seconds) mean, (SE)	0.03 (0.11)	−0.06 (0.11)	0.73
10 m walking speed (meter/second) mean, (SE)	−0.05 (1.3)	−0.081 (1.25)	0.56
400 m (seconds) mean, (SE)	0.38 (31.6)	−4.8 (44.7)	0.79
STS (seconds) mean, (SE)	0.53 (0.30	0.26 (0.27)	0.74

Results are reported as mean NfL (pg/mL) (standard error of the mean).

The table displays results from Student’s unpaired *t*-test of change from baseline to 16 week follow-up (delta values) between groups except for neurofilament light chain where results are for the main analysis using Analysis of co-variance. Lower scores indicates better performance except for SDMT and neurofilament light chain.

STS, sit-to-stand; SDMT, symbol digit modalities test; TUG, timed-up-and-go.

**TABLE 3:** Correlations between serum NfL and clinical measures.

	Rho	*P*-value
SDMT	-0.13	0.13
VO2 max	-0.06	0.52
TUG	0.06	0.48
10 m	-0.02	0.78
400 m	-0.02	0.84
STS	-0.06	0.40

The table displays results from Spearman’s rank correlation analyses between change from baseline to 16 week follow-up (delta values) in serum NfL and various cognitive and physical measures. VO2 max is results from the Åstrand test.

STS, sit-to-stand; SDMT, symbol digit modalities test; TUG, timed-up-and-go.

## Discussion

This is the first study to investigate the effects of aerobic exercise on serum NfL in patients with AD. Exercise did not reduce serum NfL. We did not find that change in NfL correlated with change in measures of cognitive function or physical fitness and function.

Previous studies of exercise effects on serum NfL have been limited to studies in multiple sclerosis or healthy participants with the exception of a single smaller study. In the study, 26 patients were randomized to either cycling or stretching but only change over time within groups were reported and not between groups. The intervention was not associated with a change over time. Ercan et al. (2021) found that in patients with relapsing-remitting multiple sclerosis, an 8-week program of moderate intensity aerobic exercise was able to decrease serum NfL levels by 32%. In another study in multiple sclerosis using a shorter exercise intervention of 3 weeks, it was similarly found that serum NfL levels were lowered (Joisten et al., 2021). These findings therefore clearly demonstrate that exercise is able to perturb processes leading to leakage of NfL even over a relatively short time scale. Joisten et al. (2021) found that the change observed in NfL in multiple sclerosis patients following exercise was linked to the kynurenine pathway which has been involved in neuroinflammatory and neurodegenerative processes (Savitz, 2020). NfL levels have been found to be associated with kynurenine pathway metabolites in early stages of AD (Chatterjee et al., 2019). Although neuroinflammation is likely to play a role in AD (Amor et al., 2010), it is reasonable to assume that this will be low-grade inflammation relative to multiple sclerosis. Exercise produces an anti-inflammatory response, but studies in mild cognitive impairment and AD have been inconsistent with some showing an effect in the direction of an anti-inflammatory effect (Tsai et al., 2019; Eustáquio et al., 2020) and others not (Nascimento et al., 2014; Jensen et al., 2019a). Further, a meta-analysis of the effect of exercise on inflammatory markers did not show an effect in AD and MCI on inflammatory markers linked to AD pathology (Huang et al., 2021). In healthy controls, exercise was also found to affect the kynurenine pathway, but not NfL (Isung et al., 2021). In summary, in AD, other drivers of pathology than neuroinflammation may be more important and less amenable to exercise, although this is speculative.

We did not find an effect of APOE genotype in terms of an effect of exercise on NfL. In contrast, in a previous analysis of results from the ADEX study, it was found that *APOE*-ε4 carriers benefitted more from the intervention on a number of cognition and physical function outcomes (Jensen et al., 2019b). Observational studies have also suggested a moderating effect of *APOE* status on the relationship between physical activity and risk of dementia, beta-amyloid status, gray and white matter volume, and cerebral blood flow, with some studies showing benefit for *APOE*-ε4 carriers (Kivipelto et al., 2008; Head et al., 2012; Brown et al., 2013; Smith et al., 2014; Zlatar et al., 2014) and others for *APOE*-ε4 non-carriers (Rovio et al., 2007; Kulmala et al., 2014; Luck et al., 2014). Evidence from differential responses in carriers versus non-carriers has also been reported in anti-amyloid therapy (Cummings et al., 2021), but it is generally assumed that this is related to amyloid removal, and so far there is no evidence to suggest that exercise modifies cortical amyloid deposition (Frederiksen et al., 2019b; Vidoni et al., 2021). It is also worth noting that it is uncertain if the difference in change in NfL is of a size that is clinically meaningful. In a large longitudinal study of changes in NfL in blood a mean change of 4.9 ng/mL was reported in AD dementia (Palmqvist et al., 2019) but whether for example halving this rate would translate into measurable effects on clinical measures remains to be determined.

Apart from a lack of an effect of exercise on neurodegeneration, other factors may explain the negative findings in the present study. For example, the intervention may not have been long enough to affect mechanisms related to the underlying disease mechanism in AD, the intervention may not have been at a sufficient intensity or the population may not have been the right one, e.g., too advanced along the AD pathophysiological pathway for exercise to be effective on NfL. A number of observations do, however, counter these explanations. Firstly, and as already mentioned, shorter interventions have been shown to be able to affect serum NfL (Ercan et al., 2021; Joisten et al., 2021), although not in AD patients. Secondly, the intervention was able to improve symptoms of AD including cognitive function (Hoffmann et al., 2016). Lastly, effects of exercise on NfL may be short-lived and were not captured due to the time from the intervention ended and blood samples were obtained. We did not find the intervention to improve measures of physical function and fitness, as opposed to findings in the whole cohort (Sobol et al., 2016). This was quite surprising and may also have played a role in the negative findings in the present study as an effect on NfL could be mediated through an improvement in, e.g., VO2max. In summary, although it cannot be ruled out, several observations may be taken to indicate that the intervention should have been able to attenuate the neurodegenerative processes, but studies with longer interventions are needed to test this.

This study has limitations. As already addressed, the length of the intervention could play a role in our negative findings. Further, it may be speculated that the intervention would have been more effective in earlier disease stages. However, finding treatments for patients at the dementia stage remains important. Another limitation is the absence of measures of other markers of neuroinflammation (or candidate markers) whose effects might be studied along with NfL.

Strengths of the study are a rigorous methodology and a relatively large sample size. Moreover, the patient population was well characterized, and all patients had a diagnosis of AD based on published criteria and assessed by experienced clinicians. The intervention was well-planned and designed specifically to patients with dementia ensuring target engagement in terms of physical fitness. In addition, all exercise sessions were supervised by a physical therapist and all participating subject wore pulse watch monitors to ensure that workout load was maintained.

In conclusion our findings do not support an effect of a 16-week intervention of moderate aerobic exercise on a single measure of neurodegeneration. Results regarding exercise in patients with AD are still sparse and although the findings do not support an effect, caution is needed, and the results do not dispel a possible disease-modifying effect of exercise. Studies examining effects on markers of AD and neurodegeneration in CSF are needed to further complement the present findings. Further studies are needed in AD examining longer interventions and applying a multimodal approach to untangle underlying mechanisms of exercise as a possible therapy in AD.

## Data availability statement

The datasets presented in this article are not readily available because data cannot be shared due to legal restrictions outside Denmark. Requests to access the datasets should be directed to KF, kristian.steen.frederiksen@regionh.dk.

## Ethics statement

The studies involving human participants were reviewed and ADEX trial was approved by the Committees of Biomedical Research Ethics for the Capital Region (protocol no.: H-3-2011-128) and the Danish Data Protection Agency (file no.: 30-0718) and carried out in accord with the Helsinki Declaration of 1975. The patients/participants provided their written informed consent to participate in this study.

## Author contributions

KF drafted the initial version of the manuscript. All authors contributed and revised the manuscript and approved the submitted version.

## References

[B1] Amor S. Puentes F. Baker D. van der Valk P. (2010). Inflammation in neurodegenerative diseases. *Immunology* 129 154–169. 10.1111/j.1365-2567.2009.03225.x 20561356 PMC2814458

[B2] Belarbi K. Burnouf S. Fernandez-gomez F. Laurent C. Lestavel S. Figeac M. (2011). Neurobiology of disease beneficial effects of exercise in a transgenic mouse model of Alzheimer’s disease-like Tau pathology. *Neurobiol. Dis.* 43 486–494. 10.1016/j.nbd.2011.04.022 21569847

[B3] Broadhouse K. M. Singh M. F. Suo C. Gates N. Wen W. Brodaty H. (2020). Hippocampal plasticity underpins long-term cognitive gains from resistance exercise in MCI. *NeuroImage Clin.* 25:102182. 10.1016/j.nicl.2020.102182 31978826 PMC6974789

[B4] Brown B. M. Peiffer J. J. Taddei K. Lui J. K. Laws S. M. Gupta V. B. (2013). Physical activity and amyloid-β plasma and brain levels: Results from the Australian imaging, biomarkers and lifestyle study of ageing. *Mol. Psychiatry* 18 875–881. 10.1038/mp.2012.107 22889922

[B5] Chatterjee P. Zetterberg H. Goozee K. Lim C. K. Jacobs K. R. Ashton N. J. (2019). Plasma neurofilament light chain and amyloid-β are associated with the kynurenine pathway metabolites in preclinical Alzheimer’s disease. *J. Neuroinflammation* 16 1–12. 10.1186/s12974-019-1567-4 31601232 PMC6788092

[B6] Cink R. Thomas T. (1981). Validity of the astrand-ryhming nomogram for predicting maximal oxygen intake. *Br. J. Sport. Med.* 15 182–185. 10.1136/bjsm.15.3.182 7272663 PMC1858756

[B7] Colcombe S. J. Erickson K. I. Scalf P. E. Kim J. S. Prakash R. McAuley E. (2006). Aerobic exercise training increases brain volume in aging humans. *J. Gerontol. A Biol. Sci. Med. Sci.* 61A 1166–1170. 10.1093/gerona/61.11.1166 17167157

[B8] Cummings J. Lee G. Zhong K. Fonseca J. Taghva K. (2021). Alzheimer’s disease drug development pipeline: 2021. *Alzheimers Dement.* 7 1–24. 10.1002/trc2.12179 34095440 PMC8145448

[B9] Eggermont L. H. Gavett B. E. Volkers K. M. Blankevoort C. G. Scherder E. J. Jefferson A. L. (2010). Lower-extremity function in cognitively healthy aging, mild cognitive impairment, and Alzheimer’s disease. *Arch. Phys. Med. Rehabil.* 91 584–588. 10.1016/j.apmr.2009.11.020 20382291 PMC2864645

[B10] Ercan Z. Bilek F. Demir C. F. (2021). The effect of aerobic exercise on Neurofilament light chain and glial Fibrillary acidic protein level in patients with relapsing remitting type multiple sclerosis. *Mult. Scler. Relat. Disord.* 55:103219. 10.1016/j.msard.2021.103219 34433118

[B11] Erickson K. I. Voss M. W. Prakash R. S. Basak C. Szabo A. Chaddock L. (2011). Exercise training increases size of hippocampus and improves memory. *Proc. Natl. Acad. Sci. U.S.A.* 108 3017–3022. 10.1073/pnas.1015950108 21282661 PMC3041121

[B12] Eustáquio F. G. Uba C. M. Guerra M. L. Luis R. Carlos C. J. Eef H. (2020). The mediating effect of different exercise programs on the immune profile of frail older women with cognitive impairment. *Curr. Pharm. Des.* 26 906–915. 10.2174/1381612826666200203123258 32013825

[B13] Firth J. Stubbs B. Vancampfort D. Schuch F. Lagopoulos J. Rosenbaum S. (2018). Effect of aerobic exercise on hippocampal volume in humans: A systematic review and meta-analysis. *Neuroimage* 166 230–238. 10.1016/j.neuroimage.2017.11.007 29113943

[B14] Folstein M. Folstein S. (1975). Mini-mental state” A practical method for grading the cognitive state of patients for the clinician. *J. Psychiat. Res.* 12 189–198. 10.1016/0022-3956(75)90026-6 1202204

[B15] Frederiksen K. Gjerum L. Waldemar G. Hasselbalch S. G. (2017). Effects of physical exercise on Alzheimer’s disease biomarkers: A systematic review of intervention studies. *J. Alzheimers Dis.* 61 359–372. 10.3233/JAD-170567 29154278

[B16] Frederiksen K. Gjerum L. Waldemar G. Hasselbalch S. G. (2019a). Physical activity as a moderator of alzheimer pathology: A systematic review of observational studies. *Curr. Alzheimer Res.* 16 362–378. 10.2174/1567205016666190315095151 30873924

[B17] Frederiksen K. Larsen C. T. Hasselbalch S. G. Christensen A. N. Høgh P. Wermuth L. (2018). A 16-week aerobic exercise intervention does not affect hippocampal volume and cortical thickness in mild to moderate Alzheimer’s disease. *Front. Aging Neurosci.* 10:293. 10.3389/fnagi.2018.00293 30319397 PMC6167961

[B18] Frederiksen K. Madsen K. Andersen B. B. Beyer N. Garde E. Høgh P. (2019b). Moderate- to high-intensity exercise does not modify cortical β-amyloid in Alzheimer’s disease. *Alzheimers Dement.* 5 208–215. 10.1016/j.trci.2019.04.006 31198839 PMC6556817

[B19] Galasko D. Bennett D. Sano M. Ernesto C. Thomas R. Grundman M. (1997). An inventory to assess activities of daily living for clinical trials in Alzheimer’s disease. The Alzheimer’s disease cooperative study. *Alzheimer Dis. Assoc. Disord.* 11 S33–S39. 9236950

[B20] Groot C. Hooghiemstra A. M. Raijmakers P. G. H. M. van Berckel B. N. M. Scheltens P. Scherder E. J. A. (2016). The effect of physical activity on cognitive function in patients with dementia: A meta-analysis of randomized control trials. *Ageing Res. Rev.* 25 13–23. 10.1016/j.arr.2015.11.005 26607411

[B21] Guralnik J. M. Simonsick E. M. Ferrucci L. Glynn R. J. Berkman L. F. Blazer D. G. (1994). A short physical performance battery assessing lower extremity function: Association with self-reported disability and prediction of mortality and nursing home admission. *J. Gerontol.* 49 M85–M94. 10.1093/geronj/49.2.m85 8126356

[B22] Head D. Bugg J. M. Goate A. M. Fagan A. M. Mintun M. A. Benzinger T. (2012). Exercise engagement as a moderator of the effects of APOE genotype on amyloid deposition. *Arch. Neurol.* 69 636–643. 10.1001/archneurol.2011.845 22232206 PMC3583203

[B23] Hoffmann K. Frederiksen K. S. Sobol N. A. Beyer N. Vogel A. Simonsen A. H. (2013). preserving cognition, quality of life, physical health and functional ability in Alzheimer’s disease: The effect of physical exercise (ADEX Trial): Rationale and design. *Neuroepidemiology* 41 198–207. 10.1159/000354632 24135720

[B24] Hoffmann K. Sobol N. A. Frederiksen K. S. Beyer N. Vogel A. Vestergaard K. (2016). Moderate-to-high intensity physical exercise in patients with Alzheimer’s disease: A randomized controlled trial. *J. Alzheimer’s Dis.* 50 443–453. 10.3233/JAD-150817 26682695

[B25] Huang X. Zhao X. Li B. Cai Y. Zhang S. Yu F. (2021). Biomarkers for evaluating the effects of exercise interventions in patients with MCI or dementia: A systematic review and meta-analysis. *Exp. Gerontol.* 151:111424. 10.1016/j.exger.2021.111424 34051283

[B26] Isung J. Granqvist M. Trepci A. Huang J. Schwieler L. Kierkegaard M. (2021). Differential effects on blood and cerebrospinal fluid immune protein markers and kynurenine pathway metabolites from aerobic physical exercise in healthy subjects. *Sci. Rep.* 11 1–13. 10.1038/s41598-021-81306-4 33462306 PMC7814004

[B27] Jensen C. S. Bahl J. M. Østergaard L. B. Høgh P. Wermuth L. Heslegrave A. (2019a). Exercise as a potential modulator of inflammation in patients with Alzheimer’s disease measured in cerebrospinal fluid and plasma. *Exp. Gerontol.* 121 91–98. 10.1016/j.exger.2019.04.003 30980923

[B28] Jensen C. S. Portelius E. Siersma V. Høgh P. Wermuth L. Blennow K. (2016). Cerebrospinal fluid amyloid beta and tau concentrations are not modulated by 16 weeks of moderate- to high-intensity physical exercise in patients with Alzheimer disease. *Dement. Geriatr. Cogn. Disord.* 42 146–158. 10.1159/000449408 27643858

[B29] Jensen C. S. Simonsen A. H. Siersma V. Beyer N. Frederiksen K. S. Gottrup H. (2019b). Patients with Alzheimer’s disease who carry the APOE ε4 allele benefit more from physical exercise. *Alzheimers Dement.* 5 99–106. 10.1016/j.trci.2019.02.007 31011620 PMC6461575

[B30] Joisten N. Rademacher A. Warnke C. Proschinger S. Schenk A. Walzik D. (2021). Exercise diminishes plasma neurofilament light chain and reroutes the kynurenine pathway in multiple sclerosis. *Neurol. Neuroimmunol. Neuroinflammation* 8 1–11. 10.1212/NXI.0000000000000982 33782190 PMC8054957

[B31] Kaufer D. I. Cummings J. L. Ketchel P. Smith V. MacMillan A. Shelley T. (2000). Validation of the NPI-Q, a brief clinical form of the Neuropsychiatric Inventory. *J. Neuropsychiatry Clin. Neurosci.* 12 233–239. 10.1176/jnp.12.2.233 11001602

[B32] Kivipelto M. Rovio S. Ngandu T. Kåreholt I. Eskelinen M. Winblad B. (2008). Apolipoprotein e ε4 magnifies lifestyle risks for dementia: A population-based study. *J. Cell. Mol. Med.* 12 2762–2771. 10.1111/j.1582-4934.2008.00296.x 18318693 PMC3828889

[B33] Koch W. Ehrenhaft A. Griesser K. Pfeufer A. Müller J. Schömig A. (2002). TaqMan systems for genotyping of disease-related polymorphisms present in the gene encoding apolipoprotein E. *Clin. Chem. Lab. Med.* 40 1123–1131. 10.1515/cclm.2002.197 12521230

[B34] Kulmala J. Solomon A. Kåreholt I. Ngandu T. Rantanen T. Laatikainen T. (2014). Association between mid- to late life physical fitness and dementia: Evidence from the CAIDE study. *J. Intern. Med.* 276 296–307. 10.1111/joim.12202 24444031

[B35] Li D. Reyderman L. Cohen S. Froelich L. Katayama S. Sabbagh M. (2022). Lecanemab in early Alzheimer’s disease. *N. Engl. J. Med.* 388 9–21. 10.1056/NEJMoa2212948 36449413

[B36] Livingston G. Sommerlad A. Orgeta V. Costafreda S. G. Huntley J. Ames D. (2017). Dementia prevention, intervention, and care. *Lancet* 390 2673–2734. 10.1016/S0140-6736(17)31363-6 28735855

[B37] Luck T. Riedel-Heller S. G. Luppa M. Wiese B. Köhler M. Jessen F. (2014). Apolipoprotein E epsilon 4 genotype and a physically active lifestyle in late life: Analysis of gene–environment interaction for the risk of dementia and Alzheimer’s disease dementia. *Psychol. Med.* 44 1319–1329. 10.1017/S0033291713001918 23883793

[B38] Maquet D. Lekeu F. Warzee E. Gillain S. Wojtasik V. Salmon E. (2010). Gait analysis in elderly adult patients with mild cognitive impairment and patients with mild Alzheimer’s disease: Simple versus dual task: A preliminary report. *Clin. Physiol. Funct. Imaging* 30 51–56. 10.1111/j.1475-097X.2009.00903.x 19799614

[B39] Molinuevo J. L. Ayton S. Batrla R. Bednar M. M. Bittner T. Cummings J. (2018). Current state of Alzheimer’s fluid biomarkers. *Acta Neuropathol.* 136 821–853. 10.1007/s00401-018-1932-x 30488277 PMC6280827

[B40] Nascimento C. M. C. Pereira J. R. Pires de Andrade L. Garuffi M. Ayan C. Kerr D. S. (2014). Physical exercise improves peripheral BDNF levels and cognitive functions in mild cognitive impairment elderly with different BDNF Val66Met genotypes. *J. Alzheimers Dis.* 43 81–91. 10.3233/JAD-140576 25062900

[B41] Palmqvist S. Insel P. S. Stomrud E. Janelidze S. Zetterberg H. Brix B. (2019). Cerebrospinal fluid and plasma biomarker trajectories with increasing amyloid deposition in Alzheimer’s disease. *EMBO Mol. Med.* 11 1–13. 10.15252/emmm.201911170 31709776 PMC6895602

[B42] Penedo F. J. Dahn J. R. (2005). Exercise and well-being: A review of mental and physical health benefits associated with physical activity. *Curr. Opin. Psychiatry* 18 189–193. 10.1097/00001504-200503000-00013 16639173

[B43] Podsiadlo D. Richardson S. (1991). The timed “Up & Go”: A test of basic functional mobility for frail elderly persons. *J. Am. Geriatr. Soc.* 39 142–148. 1991946 10.1111/j.1532-5415.1991.tb01616.x

[B44] Raket L. L. Kühnel L. Schmidt E. Blennow K. Zetterberg H. Mattsson-Carlgren N. (2020). Utility of plasma neurofilament light and total tau for clinical trials in Alzheimer’s disease. *Alzheimers Dement.* 12 1–9. 10.1002/dad2.12099 32995466 PMC7507310

[B45] Rovio S. Kåreholt I. Viitanen M. Winblad B. Tuomilehto J. Soininen H. (2007). Work-related physical activity and the risk of dementia and Alzheimer’s disease. *Int. J. Geriatr. Psychiatry* 22 874–882. 10.1002/gps.1755 17721898

[B46] Savitz J. (2020). The kynurenine pathway: A finger in every pie. *Mol. Psychiatry* 25 131–147. 10.1038/s41380-019-0414-4 30980044 PMC6790159

[B47] Selkoe D. J. Hardy J. (2016). The amyloid hypothesis of Alzheimer’s disease at 25 years. *EMBO Mol. Med.* 8 595–608. 10.15252/emmm.201606210 27025652 PMC4888851

[B48] Smith A. (1973). *Symbol digit modalities test. Manual.* Los Angeles, CA: Western Psychological Services.

[B49] Smith J. C. Nielson K. A. Woodard J. L. Seidenberg M. Durgerian S. Hazlett K. E. (2014). Physical activity reduces hippocampal atrophy in elders at genetic risk for Alzheimer’s disease. *Front. Aging Neurosci.* 6:61. 10.3389/fnagi.2014.00061 24795624 PMC4005962

[B50] Sobol N. A. Hoffmann K. Frederiksen K. S. Vogel A. Vestergaard K. Braendgaard H. (2016). Effect of aerobic exercise on physical performance in patients with Alzheimer’s disease. *Alzheimers Dement.* 12 1207–1215. 10.1016/j.jalz.2016.05.004 27344641

[B51] Tsai C.-L. Pai M.-C. Ukropec J. Ukropcová B. (2019). Distinctive effects of aerobic and resistance exercise modes on neurocognitive and biochemical changes in individuals with mild cognitive impairment. *Curr. Alzheimer Res.* 16 316–332. 10.2174/1567205016666190228125429 30819077

[B52] Vasconcelos-Filho F. S. L. da Rocha Oliveira L. C. de Freitas T. B. C. de Pontes P. A. D. S. da Rocha-e-Silva R. C. Godinho W. D. N. (2021). Effect of involuntary chronic physical exercise on beta-amyloid protein in experimental models of Alzheimer’s disease: Systematic review and meta-analysis. *Exp. Gerontol.* 153:111502. 10.1016/j.exger.2021.111502 34339821

[B53] Vidoni E. D. Morris J. K. Watts A. Perry M. Clutton J. van Sciver A. (2021). Effect of aerobic exercise on amyloid accumulation in preclinical Alzheimer’s: A 1-year randomized controlled trial. *PLoS One* 16:e0244893. 10.1371/journal.pone.0244893 33444359 PMC7808620

[B54] Wang R. Zhang H. Li H. Ren H. Sun T. Xu L. (2022). The influence of exercise interventions on cognitive functions in patients with amnestic mild cognitive impairment: A systematic review and meta-analysis. *Front. Public Health* 10:1046841. 10.3389/fpubh.2022.1046841 36457329 PMC9706097

[B55] Wilckens K. A. Stillman C. M. Waiwood A. M. Kang C. Leckie R. L. Peven J. C. (2021). Exercise interventions preserve hippocampal volume: A meta-analysis. *Hippocampus* 31 335–347. 10.1002/hipo.23292 33315276 PMC11497212

[B56] Zlatar Z. Z. Wierenga C. E. Bangen K. J. Liu T. T. Jak A. J. (2014). Increased hippocampal blood flow in sedentary older adults at genetic risk for Alzheimer’s disease. *J. Alzheimers Dis.* 41 809–817. 10.3233/JAD-132252 24685629 PMC4259215

